# Obesity and periodontitis: A systematic review and updated meta-analysis

**DOI:** 10.3389/fendo.2022.999455

**Published:** 2022-10-24

**Authors:** Chang Min Kim, Soobin Lee, Wonjun Hwang, Eunjeong Son, Tae Woo Kim, Kihun Kim, Yun Hak Kim

**Affiliations:** ^1^ School of Dentistry, Pusan National University, Yangsan, South Korea; ^2^ Department of Internal Medicine, Pusan National University Yangsan Hospital, Yangsan, South Korea; ^3^ Research Institute for Convergence of Biomedical Science and Technology, Pusan National University Yangsan Hospital, Yangsan, South Korea; ^4^ Department of Occupational and Environmental Medicine, Kosin University Gospel Hospital, Busan, South Korea; ^5^ Department of Biomedical Informatics, School of Medicine, Pusan National University, Yangsan, South Korea; ^6^ Department of Anatomy, School of Medicine, Pusan National University, Yangsan, South Korea

**Keywords:** obesity, periodontitis, observational study, systematic review, meta-analysis

## Abstract

**Background:**

A previous 2014 meta-analysis reported a positive association between obesity and periodontitis. It was considered necessary to update the recently published papers and to analyse subgroups on important clinical variables that could affect the association between obesity and periodontitis. Therefore, we updated the latest studies and attempted to derive more refined results.

**Methods:**

All observational studies were eligible for inclusion. The Newcastle–Ottawa scale was used to qualitatively evaluate the risk of bias. Subgroup analyses were conducted for patients aged 18–34, 35–54, and 55+ years and the countries (European countries, USA, Brazil, Japan, Korea, and other Asian countries).

**Results:**

Thirty-seven full-text articles were included. Obesity conferred increased odds of periodontal disease with an odds ratio (1.35, 95% CI: 1.05–1.75). In the subgroup analysis by age, the odds ratio was the highest in the 18–34 years group (2.21, 95% CI: 1.26–3.89). In the subgroup analysis by country, European countries had the highest odds ratio (2.46, 95% CI: 1.11–5.46).

**Conclusion:**

Despite the differences in degree, a positive association between obesity and periodontitis was found regardless of country or age. Therefore, medical professionals should try to prevent periodontitis by controlling patient weights, and more studies should be conducted to determine the association between obesity and oral health.

**Systematic Review Registration:**

https://www.crd.york.ac.uk/prospero/, identifier CRD42022301343.

## Introduction

Obesity has increased worldwide in the past 50 years and become a significant social problem (1). The prevalence of obesity is seen in one-third of the entire population, and it increases in all age groups of both sexes (2). According to the World Health Organization, which defines obesity as a body mass index (BMI) ≥ 30 kg/m^2^, obesity is a 21^st^-century epidemic and health risk factor with a prevalence that is rapidly increasing in children and adolescents (3, 4).

Obesity negatively impacts an individual’s physical and mental health, leading to poor quality of life (5). Obesity can aggravate chronic inflammatory diseases such as diabetes and coronary artery disease (6). Obesity also significantly affects cancer risk and prognosis in individuals (7). Adipokines, which are found in obesity, may induce inflammation, atherosclerosis, diabetes, and psoriasis (8). Patients with coronavirus disease 2019 and who are obese showed higher risks and had worse outcomes than those who were not obese (9). It has been reported that obesity has negative effects on health and systemic diseases (10).

Periodontitis is a very common disease with a high global prevalence as half of all adults worldwide have at least one tooth with apical periodontitis (11). According to the National Health and Nutrition Examination Survey of adults in the US (2009–2014), it is seen that periodontitis is highly prevalent in adults 30 years or older (12). Its social burden has been increasing globally, warranting global changes in public health policy (13). However, more studies related to its risk factors are needed to prevent it.

Several studies have reported an association between obesity and periodontitis. A previous meta-analysis conducted in 2014 reported a positive association between the two diseases (14). It was thought necessary to update the recently published papers and to analyze subgroups on clinical variables (e.g., age or country) that could affect the association between obesity and periodontitis. Therefore, the current study aimed to include newly published studies and to perform novel subgroup analyses.

## Methods

### Information sources and search strategy

This systematic review was conducted in accordance with the PRISMA guidelines (15). This guideline was designed to allow authors to report transparently why this review was done, what they did, and what they found. PRISMA checklist was presented in Supplementary materials. The study protocol was registered with PROSPERO (registration number: CRD42022301343). We searched the Embase and PubMed (Medline) databases for studies published between 2010 and January 3, 2022. Initially, the mesh term was considered when establishing a search strategy. However, it was not sufficient to search for relevant papers with only mesh terms. A search strategy was established by adding mesh terms as well as free words related to the topic through discussion between authors. The search strategy was presented in **Supplementary Table 1**. The search screened the titles and abstracts. Non-human studies, non-articles, and conference abstracts were excluded.

### Eligibility criteria

Studies investigating periodontitis in obese and normal-weight individuals of all ages were also included. A previous meta-analysis article that synthesised studies published through 2010 was included, and studies published after 2010 were searched and selected (14). Odds ratio (OR) was identified for studies that included four groups according to the state of periodontitis and obesity. All observational studies (cohort, case-control, and cross-sectional studies) were eligible for inclusion. Case reports, review articles, animal studies, and studies without a control group were excluded. In the case of duplicated studies, those with a larger range of patients were included, while those that did not were excluded. Among the studies in the previous meta-analysis, cases in which the data were incorrectly extracted were confirmed and excluded. Only cases in which periodontitis and obesity were classified according to clear criteria were included. We defined obesity is as a BMI ≥ 30kg/m^2^ or higher, but BMI ≥ 25 kg/m^2^ in Asians (16, 17). In addition, We additionally defined obesity based on waist circumference as ≥88 cm in women and ≥102 cm in men, but ≥ 90 cm for men and ≥ 80 cm for women in Asians (18, 19). Periodontal status was based on the periodontal pocket depth, clinical attachment loss, or community periodontal index. Periodontitis was defined as periodontal pocket depth (PPD) ≥ 4mm, clinical attachment level (CAL) ≥ 1mm, and community periodontal index (CPI) ≥ 3 (20, 21). We excluded papers that did not meet the obesity criteria (e.g., BMI mean) or periodontitis criteria.

### Study selection and data extraction processes

The literature search was conducted independently by three authors (CK, SL, and WH) who thoroughly screened the titles and abstracts of each study. The full-text articles were reviewed by the same authors and evaluated for eligibility. Any disagreements were resolved through discussion. The extracted information included the number of patients, mean age and range, sex, country providing the sample, periodontal disease evaluation method, nutritional status evaluation method, and main confounding variables. We extracted the number of samples or ORs as effect measures according to all patient and age groups for the data synthesis and subgroup analysis.

### Statistical methods

We performed meta-analyses to calculate the pooled odds ratio and corresponding 95% confidence intervals (CIs) stratified according to obesity and periodontitis status. The classification of I^2^ statistics as presented by Higgins et al. was used to evaluate the heterogeneity of the effect measures (22). Heterogeneity was considered low, moderate, or high for I^2^ values of 25%, 50%, or 75%, respectively. An I² value > 50% indicated substantial heterogeneity. If the heterogeneity exceeded 50%, the random effects method was used; otherwise, the fixed effects method was used. We considered the results statistically significant at values of *p* < 0.05 or when the CI did not include 1. Review Manager 5.4 software was used to analyse the results. Forest plots were drawn to clearly visualise the synthesised risk. Subgroup analyses were conducted for patients aged 18–34 years, 35–54 years, and 55+ years as well as the countries providing the samples (European countries, USA, Brazil, Japan, Korea, and other Asian countries).

### Risk of bias within studies

The Newcastle–Ottawa scale was used to qualitatively evaluate the risk of bias for cohort and case-control studies (23). The adapted version of the Newcastle–Ottawa scale presented by Herzog et al. (24) was used to evaluate cross-sectional studies (24). We evaluated the score for each category and classified it as good, fair, and poor according to the Agency for Healthcare Research and Quality (AHRQ) standard (25). We evaluated study quality by establishing a criterion similar to the AHRQ standard for cross-sectional studies.

### Publication bias

A funnel plot was drawn to visually evaluate publication bias using Review Manager 5 (RevMan 5). Egger’s regression test was performed to statistically verify publication bias using Stata 13 software.

### Certainty assessment

We used the GRADE approach, a tool for measuring the overall grade in risk estimates as high, moderate. low, or very based on 8 classifications; study limitation, directness, consistency, precision, reporting bias, dose-response association, plausible confounding that would decrease observed effect, and strength of association (26, 27). The assessment tools were shown in **Supplementary Tables 2** and **3**.

## Results

### Study selection and characteristics

**Figure 1:**
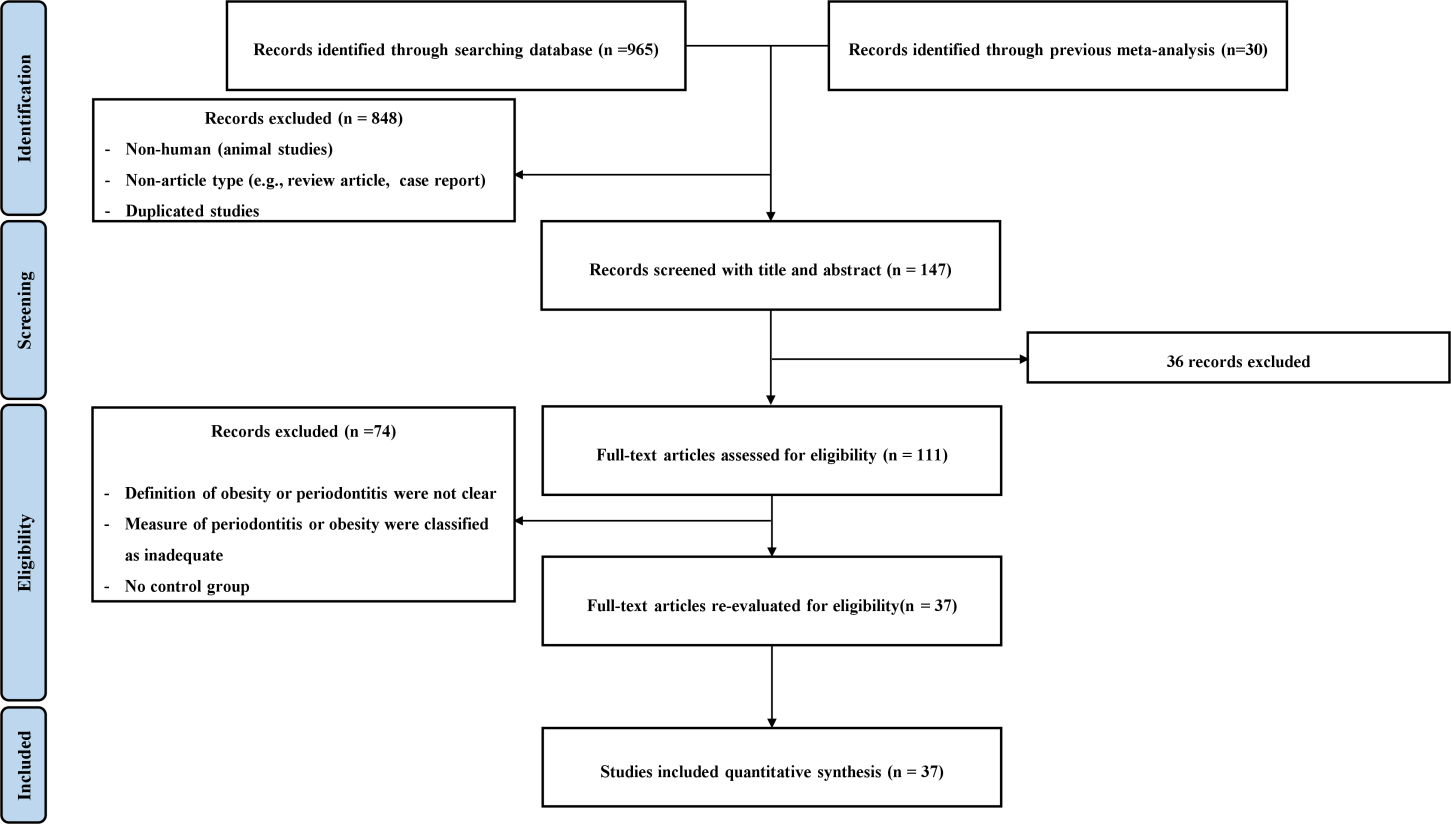
PRISMA flow diagram.

A total of 995 records, including 30 studies from the previous meta-analysis study and 965 studies, were initially found based on the search terms. We excluded 848 records based on the exclusion criteria, including animal studies, duplicated studies, irrelevant articles; therefore, we fully screened 147 records. Of those, 36 studies, which were unrelated to our study topic, were excluded. Thus, a full-text review of 111 papers was conducted. We excluded 74 papers according to the following criteria: inadequate measures of periodontitis or obesity classification or no control group. Ultimately, 37 full-text articles were assessed and finally included (**Figure 1**). The characteristics of the included studies are shown in **Table 1**.

**Table 1:** Characteristics of the included studies.

Reference(Author, year)	Subjects(n)	Age range	Mean age(years)	Percent female(%)	Country	Method of periodontal evaluation and criteria	Method of obesity evaluation and criteria	Main confounding variables	Significant association
**(** 28 **)**	13665	≥ 18	NR	52.70%	USA	PPD ≥ 4mm and CAL ≥ 3mm	BMI ≥ 30Kg/m²	Age, gender, ethnicity, smoking habits, diabetes, schooling, last dental visit	Yes
**(** 29 )*	186	35~64	54	45.20%	France	PPD ≥ 4mm and CAL ≥ 4mm	BMI ≥ 30kg/m²	age, gender, education level, smoking habits, physical activity, energy intake, C-reactive protein, high-carbohydrate’ diet, insulin	Yes
**(** 30 **)**	365	> 60	73	NR	Mexico	PPD ≥ 6mm	BMI ≥ 30Kg/m²	age, gender, sociodemographic factors, schooling, smoking habits	No
**(** 31 )*	12420	≥ 20	NR	66.83%	Taiwan	CAL ≥ 1mm	BMI ≥ 30kg/m²	Age, Sex, Monthly income	Yes
**(** 32 **)**	13677	> 17	NR	NR	USA	PPD ≥ 4mm	WC > 102cm (male) or WC > 88cm(female)	Age, gender, smoking habits, schooling, ethnicity	Tendency
**(** 33 **)**	706	30~65	NR	53.40%	Brazil	CAL ≥ 5mm	BMI ≥ 30Kg/m²	Diabetes	Yes
**(** 34 **)**	79	19~69	NR	60.80%	Norway	PPD > 6mm	BMI ≥ 30Kg/m²	None	Yes
**(** 35 **)**	618	18~24	21.4	52.10%	Japan	CPI scores 3 and 4	BMI ≥ 30Kg/m²	Unclear	Yes
**(** 36 **)**	2225	18~19	18.6	43.20%	Japan	PPD ≥ 4mm	BMI ≥ 25Kg/m²	None	Tendency
**(** 37 )*	539	≥ 18	45	17.99%	Brazil	PPD ≥ 4mm and CAL ≥3mm	BMI ≥ 30kg/m²	age, sex, smoking, alcohol consumption	Tendency
**(** 38 )*	400	50~75	62.5	100%	Jordan	CPI scores ≥ 3 and PPD ≥ 4mm	BMI ≥ 30kg/m²	BMI, BMD, educational level, income level, parity, employment	Yes
**(** 39 **)**	695	18~86	46.8	49.60%	USA	PPD ≥ 4mm	BMI ≥ 30Kg/m²	Antibiotic therapy, age, gender, smoking habits	Yes
**(** 40 **)**	1046	15~84	40.8	0.545	Korea	CPI scores ≥ 3	BMI ≥ 25kg/m²	age, gender, monthly family income, smoking, drinking, frequency of daily teeth brushing, physical activity	Yes
**(** 41 )*	168	18~60	38.2	76.19%	Jordan	CAL ≥ 3mm	BMI ≥ 30kg/m²	Age, Gender, Educational level, BMI	Yes
**(** 42 )*	197	25~40	NR	44.20%	Japan	CPI scores ≥ 3	BMI ≥ 25kg/m²	Unclear	Yes
**(** 43 **)**	340	18~70	NR	50.60%	Jordan	PPD ≥ 4mm and CAL ≥ 3mm	BMI ≥ 30Kg/m² and WC > 102cm (men) or > 88cm (women)	Pregnancy, antibiotic therapy, osteoporosis, cancer, age, dental plaque, number of teeth	Yes
**(** 44 )*	11466	≥ 18	NR	57.66%	Korea	CPI scores 3 and 4	BMI ≥ 25kg/m²	gender, age, educational level, household income, smoking, frequency of tooth brushing, diabetes mellitus	Tendency
**(** 45 )*	36110	40~65	NR	63.40%	Japan	CPI scores 3 and 4	BMI ≥ 30kg/m²	age, sex, number of PT	Tendency
**(** 46 **)**	1504	20~95	52.8	53.90%	Denmark	CAL ≥ 3mm	BMI ≥ 30Kg/m²	Age, gender, smoking habits, diabetes, physical activity	No
**(** 47 **)**	513	18~54	32.6	NR	India	CPI scores 3 and 4	BMI ≥ 30Kg/m²	Age	Yes
**(** 48 **)**	1070	40~70	NR	73.70%	Japan	CPI score 4	BMI ≥ 25Kg/m²	Age, gender, smoking habits	No
**(** 49 )*	354850	40~79	NR	39.38%	Korea	PPD ≥ 4mm and CAL ≥ 3mm	BMI ≥ 30kg/m²	sex, age, household income, insurance status, residence area, health status, smoking status	Tendency
**(** 50 **)**	208	37~78	61.1	54.80%	China	CAL ≥ 3mm	BMI ≥ 25Kg/m² and WC > 90cm (men) or > 80cm(women)	Antibiotic therapy, age, gender, smoking habits	Tendency
**(** 51 **)**	60	> 20	43.9	43.30%	Brazil	PPD ≥ 5mm	BMI ≥ 30Kg/m²	age, gender	No
**(** 52 )*	125	11~18	14.8	52.80%	Belgium	PPD ≥ 4mm	BMI ≥ 30kg/m²	Age, Gender	No
**(** 53 )*	212	20~65	NR	41.98%	Spain	PPD ≥ 4mm and CAL ≥ 3mm	BMI ≥ 30kg/m²	Unclear	Yes
**(** 54 **)**	2478	24~60	43.3	18.20%	Japan	CPI scores 3 and 4	BMI ≥ 25Kg/m²	age, gender, smoking habits	Yes
**(** 55 )*	1619	20~56	39.7	20.57%	Japan	CPI scores 3 and 4	BMI ≥ 25kg/m²	Age, Gender, Toothbrushing frequency, Smoking habit, Hyperglycemia, Dyslipidemia, Hypertension	Tendency
**(** 56 )*	594	18~65	39.7	100%	Brazil	PPD ≥ 5mm and CAL ≥ 4mm	BMI ≥ 30kg/m²	age, education, martial status, smoking habits, diabetes, hypertension, dyslipidemia	Yes
**(** 57 )*	367	≥ 18	34.9	59.67%	Vietnam	PPD ≥ 5mm	BMI ≥ 25kg/m²	demographic characteristics, dental behaviors, self-perception of oral status, and dental knowledge	Yes
**(** 58 **)**	643	NR	45.6	79.60%	Japan	PPD ≥ 4mm	BMI ≥ 30Kg/m²	age, gender, social class, diabetes, smoking habits, oral hygiene	Yes
**(** 59 **)**	76	55~59	55	100.00%	Japan	PPD 3 teeth ≥ 4mm or 1 tooth ≥ 6mm	BMI ≥ 25Kg/m²	Gender	Yes
**(** 60 **)**	214	30~59	44.3	58.40%	Finland	PPD ≥ 4mm	BMI ≥ 30Kg/m²	age, gender, schooling, dental plaque, number of teeth	Tendency
**(** 61 )*	1160	20~77	NR	NR	Japan	CPI scores ≥ 3	BMI ≥ 30kg/m²	age, gender, number of teeth, smoking status, fasting plasma glucose, systolic blood pressure	Tendency
**(** 62 )*	863	≥ 18	NR	0%	Japan	CPI scores 3 and 4	BMI ≥ 25Kg/m²	eating speed, number of missing functional teeth, periodontal status, age, military ranks, alcohol habit, smokng, exercise	Yes
**(** 63 **)**	2005	50~73	60	25.60%	Thailand	CAL ≥ 4mm	BMI ≥ 25Kg/m²	age, dental plaque, smoking habits, diabetes	No
**(** 64 **)**	12123	35~44	39.5	64.30%	Taiwan	CPI scores 3 and 4	BMI ≥ 25Kg/m²	severe systemic disease	Tendency

BMI, body mass index; CAL, clinical attachment level; CPI, community periodontal index; NR, not reported; PPD, periodontal pocket depth; WC, waist circumference

*Newly included in this study.

### Synthesis of results

#### Overall obesity–periodontitis association

**Figure 2:**
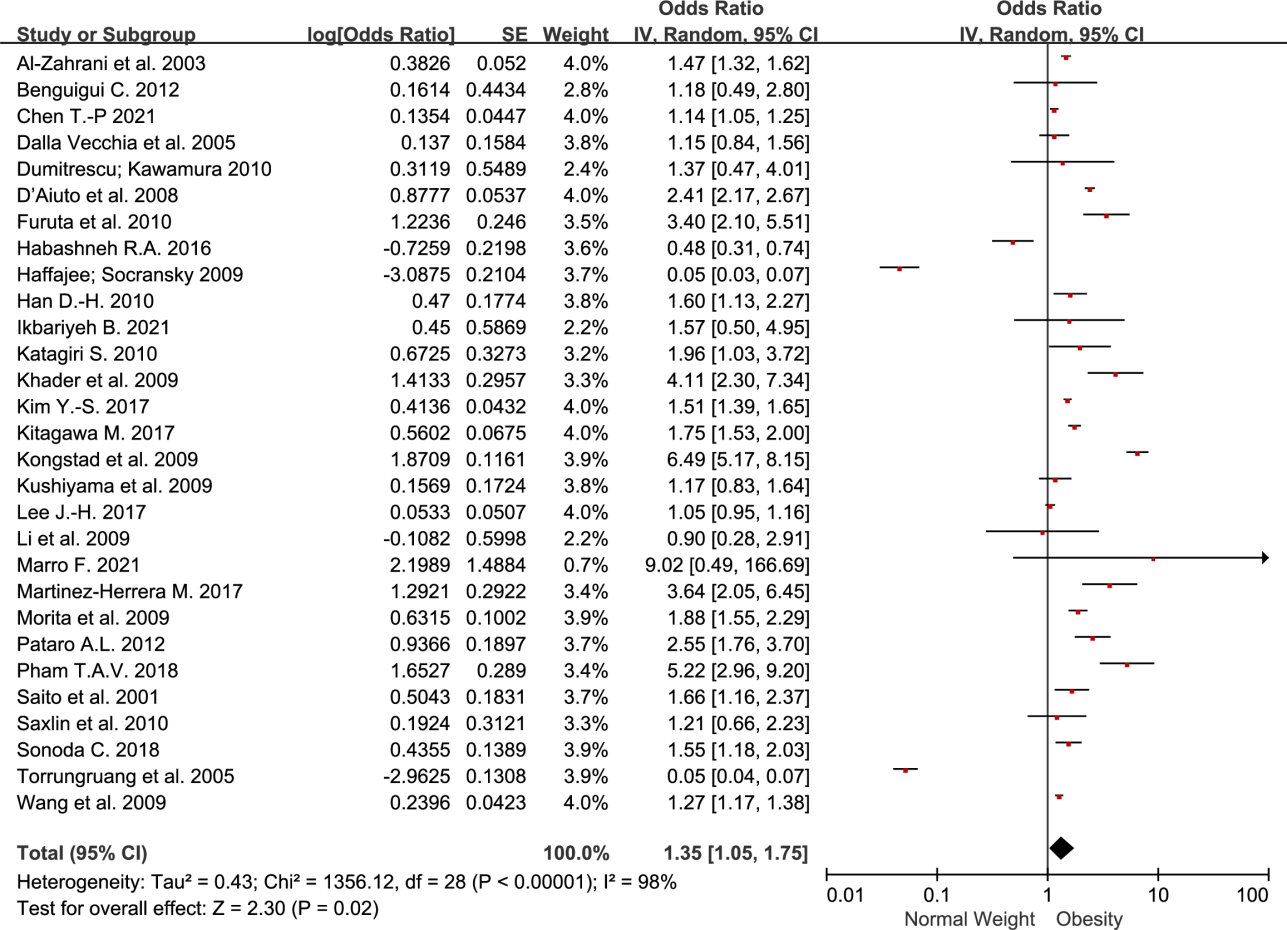
Forest plot of the risk of periodontitis for obesity group compared to non-obesity group.

We included 29 studies specifying the number of BMI–periodontitis cases or ORs. Among them, the risk of periodontitis was positively associated with obesity in 17 studies. Compared with non-obese group, obesity group conferred increased odds of periodontal disease with an OR of 1.35 (1.05-1.75) (**Figure 2**). The heterogeneity was 98%.

#### Obesity-periodontitis association stratified by age

To determine the effect of age on the obesity-periodontitis association, we classified patients into 18–34, 35–54, and 55+ years age groups. The OR was 2.21 (1.26–3.89) in 18–34 years group, 1.53 (1.17–2.00) in 35–54 years group, and 1.82 (1.16–2.83) in 55+ years group (**Table 2**).

**Table 2:** Subgroup analysis stratified by age and country.

Subgroup analysis	Number of included studies	Heterogeneity (%)	Odds ratio (95% confidence interval)
Age			
18–34 years	4	58	2.21 (1.26-3.89)
35–54 years	6	84	1.53 (1.17-2.00)
55+ years	4	78	1.82 (1.16-2.83)
Country			
USA	3	99	0.59 (0.19-1.65)
Brazil	2	90	1.70 (0.78-3.72)
European countries	6	88	2.46 (1.11-5.46)
Korea	3	93	1.34 (1.00-1.80)
Japan	7	58	1.75 (1.48-2.06)
Other Asian countries	8	99	0.98 (0.49-1.95)

#### Obesity-periodontitis association stratified by country

To evaluate the association by country, we classified the patients into country groups (European countries, USA, Brazil, Korea, Japan, and other Asian countries) and compared their results (**Table 2**). The largest OR was noted in European countries 2.46 (1.11-5.46). The OR was 0.59 (0.19-1.65) in the USA and 1.70 (0.78-3.72) in Brazil. The odds ratio was 1.34 (1.00-1.80) in Korea, 1.75 (1.48-2.06) in Japan, and 0.98 (0.49-1.95) in other Asian countries.

### Risk of bias within studies

We assessed the quality of the included studies using the Newcastle–Ottawa scale. Of the 29 cross-sectional studies, 21 were evaluated as ‘very good’ and 8 were evaluated as ‘good’ (**Supplementary Table 4**). Of the 4 cohort studies, 3 were evaluated as ‘good’ and 1 as ‘fair’ (**Supplementary Table 5**). Of the 4 case-control studies, 3 were evaluated as ‘good’ and 1 as ‘fair’ (**Supplementary Table 6**).

### Publication bias across studies

**Figure 3:**
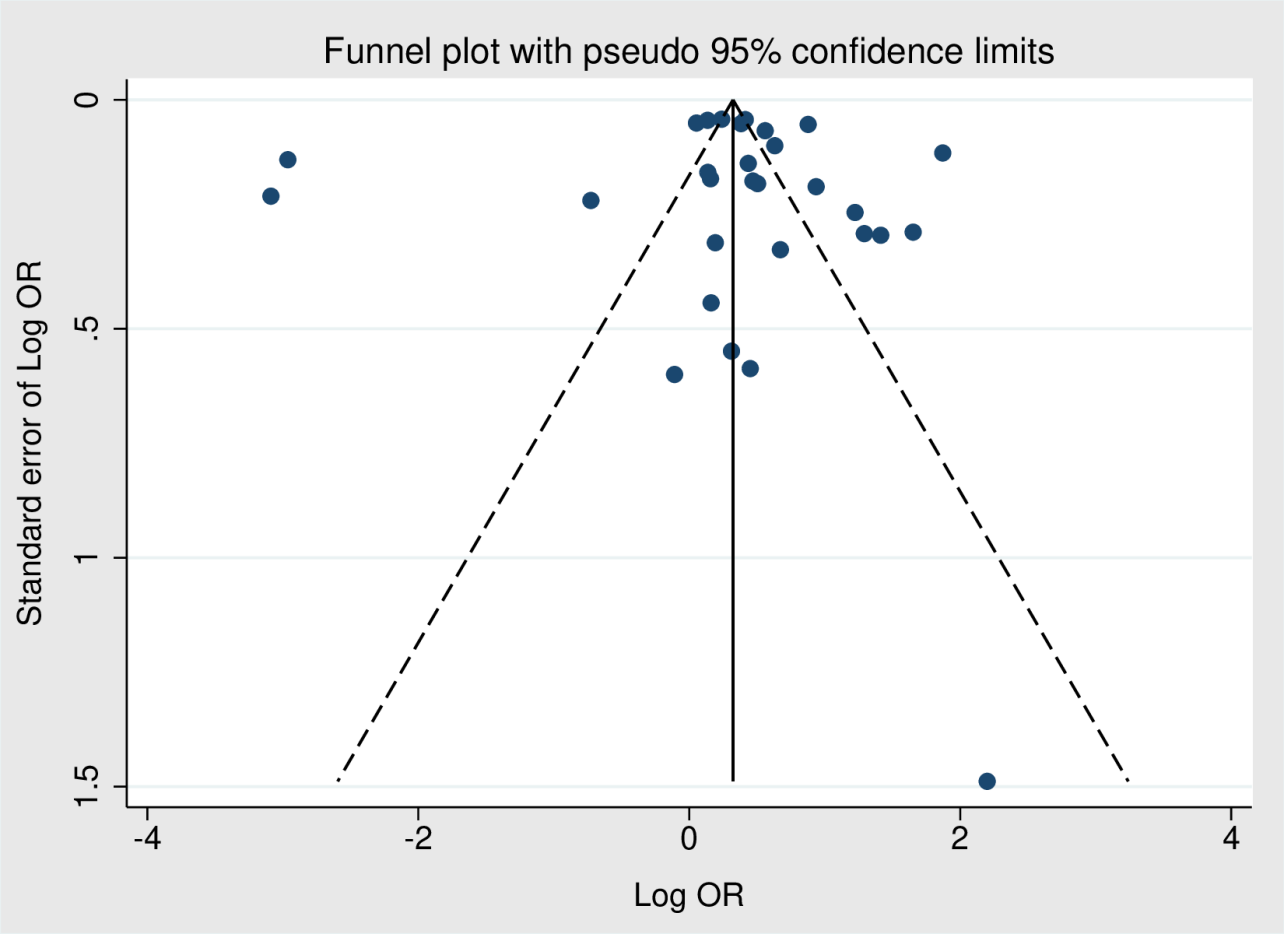
Funnel plot for the risk of periodontitis for obesity group compared to non-obesity group.

A funnel plot for the overall obesity-periodontitis association is shown in **Figure 3**. Egger’s regression test revealed no significant publication bias (*p* = 0.871).

### Certainty assessment

The strength of evidence for 8 domains was rated individually for the primary outcome. The quality of evidence was low according to the GRADE approach (**Table 3**).

**Table 3:** GRADE approach for the primary outcome.

	Quality assessment								
Outcome	Required domains					Additional domains			Grade
		Study limitations	Consistency	Directness of evidence	Precision	Reporting bias	Dose-response association	Plausible confounding that would decrease observed effect		Strength of association (magnitude of effect)	
**Periodontitis**	**High^a^**	**Inconsistent^b^**	**Indirect**	**Precise^c^**	**Undetected^d^**	**Undetected**	**Present^e^**	**Weak^f^**	**⨁◯◯◯ Very low**

^a^All included studies are observational design.

^b^Considerable heterogeneity (I^2 =^ 97%).

^c^Very large sample size (over 4,000).

^d^According to Egger’s regression test (p=0.907).

^e^All included studies are observational design, and adjusted analysis was performed differently for each study.

^f^OR=1.35.

## Discussion

This systematic review and meta-analysis provided strong evidence to support the positive association between obesity and periodontitis. Among the 29 studies, 17 studies showed a significant increased odds ratio of periodontitis in the obesity group. Nine studies showed not statistically significant results, and 3 studies showed statistically decreased odds ratio.

As periodontitis progresses differently by age, it is necessary to focus on its association with age (65). The OR of the 18–34 years group was 2.21 (1.26–3.89), which showed the highest association between obesity and periodontitis. Young people have better oral health than other age groups (66). Thus, they have fewer risk factors for periodontitis, and the impact of each risk factor is higher. Therefore, periodontitis is greatly affected by obesity, a risk factor in a large proportion of young people. The OR of the 35–54 years group was 1.53 (1.17–2.00) and 55+ years group was 1.82 (1.16–2.83). This is an expected result indicating that elderly individuals are more vulnerable to periodontitis than middle-aged individuals. Therefore, they are vulnerable to the effects of obesity (67). In conclusion, both elderly and young people showed a significant correlation between obesity and periodontitis. Why is the OR value for young people noticeably high? Young people with few other periodontitis risk factors are largely affected by obesity. In addition, especially vulnerable groups (e.g., autism spectrum disorder) among young people may be more susceptible to oral pathogens due to poor oral hygiene management and preference for cariogenic foods (68, 69). Therefore, constant weight management and inspection are needed to maintain the periodontal health of young people.

Compared to the overall OR in the present study, European countries had highest OR among all countries. High odds ratio observed in European countries also could be explained by several factors. According to Nazir et al. (70), the high prevalence of periodontal disease in European countries may be attributed to the high proportion of older population and easy access to medical institutions (70). Japan had a high OR among Asian countries. This might be partially explained by the Japanese medical system. Because Japan has universal health insurance systems, the economic barrier to dental care would be low (71). In addition, the coverage and amount of cost are wider than Korea, which has similar universal health insurance systems (71). Therefore, it was thought that high odds ratio would be observed because the accessibility of dental care may be higher than other countries when periodontitis occurs.

Our study has several limitations. It is difficult to identify a causal relationship between obesity and periodontitis because all included study designs were observational. There were differences in the definition of obesity and periodontitis in each study, which may lead to selection bias. No adjustment was performed for potential confounders such as diabetes, diet, and smoking habit. The true value ​​could be distorted by these confounding factors, and there is a possibility of toward null or away from null depending on the nature of the confounder (72). The search was performed using only the Embase and Medline databases. Nevertheless, it is thought that this meta-analysis has the advantage of updating the latest studies to increase external validity and drawing more precise conclusions through subgroup analysis.

## Conclusion

European countries and Japan showed a significant positive association, and the USA, Brazil, and other Asian countries showed insignificant association. A positive association was found regardless of age. Therefore, medical professionals should try to prevent periodontitis by controlling patient weights, and more studies should be conducted to determine the association between obesity and oral health.

## Data availability statement

The original contributions presented in the study are included in the article/**Supplementary Material**. Further inquiries can be directed to the corresponding authors.

## Author contributions

CK: Methodology, Investigation, Formal analysis, Data curation, Writing – Original draft preparation. SL: Methodology, Investigation, Formal analysis, Data curation, Writing – Original draft preparation. WH: Methodology, Investigation, Formal analysis, Data curation, Writing – Original draft preparation. ES: Software, Visualization, Supervision. TK: Software, Visualization, Supervision. KK Conceptualization, Visualization, Project Administration, Writing – Reviewing and Editing, Supervision. YK: Conceptualization, Visualization, Project Administration, Funding acquisition, Writing – Reviewing and Editing, Supervision. All authors contributed to the article and approved the submitted version.

## Funding

This work was supported by the Medical Research Center (MRC) program [grant number NRF-2018R1A5A2023879] and the Basic Science Research Program [grant number NRF-2020R1C1C1003741], and the Ministry of Health & Welfare, Republic of Korea (HI22C1377).

## Conflict of interest

The authors declare that the research was conducted in the absence of any commercial or financial relationships that could be construed as a potential conflict of interest.

## Publisher’s note

All claims expressed in this article are solely those of the authors and do not necessarily represent those of their affiliated organizations, or those of the publisher, the editors and the reviewers. Any product that may be evaluated in this article, or claim that may be made by its manufacturer, is not guaranteed or endorsed by the publisher.

## Abbreviations

AHRQ, Agency for Healthcare Research and Quality; BMI, body mass index; CIs, confidence intervals; OR, Odds ratio.
