# Supplementary material for: Obesity and periodontitis: A systematic review and updated meta-analysis
Source: Front Endocrinol (Lausanne). 2022 Oct 24;13:999455. doi: 10.3389/fendo.2022.999455 (PMC9637837; doi:10.3389/fendo.2022.999455)
Supplement: Supplementary file 1 [file Table_1.docx]

**Supplementary Table 1. Search Strategy**

| Search categories | Search terms (combined with AND) |
| --- | --- |
| Obesity | Obesity:ab,ti [MeSH Terms] or overweight:ab,ti [MeSH Terms] or body weight:ab,ti [MeSH Terms] or body mass index:ab,ti [MeSH Terms] or abdominal fat:ab,ti [MeSH Terms] or obese:ab,ti [Text Word] or BMI:ab,ti [Text Word] |
| Periodontitis | periodontitis:ab,ti [MeSH Terms] or periodontal disease:ab,ti [MeSH Terms] or gingivitis:ab,ti [MeSH Terms] or oral health:ab,ti [MeSH Terms] or oral hygiene:ab,ti [MeSH Terms] or tooth loss:ab,ti [MeSH Terms] or missing teeth:ab,ti [MeSH Terms] or oral disease:ab,ti [Text word] or periodontal inflammation:ab,ti [Text word] or gum disease:ab,ti [Text word] or gum inflammation:ab,ti [Text word] or parodontitis:ab,ti [Text word] or paradentosis:ab,ti [Text word] |
| Outcome | risk:ab,ti [MeSH Terms] or prevalence:ab,ti [MeSH Terms] or incidence:ab,ti [MeSH Terms] or outcome:ab,ti [Text word] or prognosis:ab,ti [MeSH Terms] or morbidity:ab,ti [MeSH Terms] or ratio:ab,ti [Text word] or hazard:ab,ti [Text word] or odds:ab,ti [Text word] |

**Supplementary Table 2. Full form of GRADE approach**

| **Domain** | **Definition and elements** | **Score and application** |
| --- | --- | --- |
| Study limitations | Study limitations is the degree to which the included studies for a given outcome have a high likelihood of adequate protection against bias | Low / Medium / High |
| Directness | Directness relates to (a) whether evidence links interventions directly to a health outcome of specific importance for the review, and (b) for comparative studies, whether the comparisons are based on head-to-head studies. | Direct / Indirect |
| Consistency | Consistency is the degree to which included studies find either the same direction or similar magnitude of effect. | Consistent / Inconsistent / Unknown |
| Precision | Precision is the degree of certainty surrounding an effect estimate with respect to a given outcome, based on the sufficiency of sample size and number of events. | Precise / Imprecise |
| Reporting bias | Reporting bias results from selectively publishing or reporting research findings based on the favorability of direction or magnitude of effect. | Suspected / Undetected |
| Dose-response association | This domain should be considered when studies in the evidence base have noted levels of exposure (dose, duration, adherence) | Present / Undetected |
| Plausible confounding that would decrease observed effect | Occasionally, in an observational study, plausible confounding would work in the direction opposite that of the observed effect. Had these confounders not been present, the observed effect would have been even larger than the one observed. | Present / Absent |
| Strength of association (magnitude of effect) | Strength of association refers to the likelihood that the observed effect is large enough that it cannot have occurred solely as a result of bias from potential confounding factors. | Strong / Weak |

**Supplementary Table 3. Strength of evidence grades and definitions for GRADE approach**

| **Grade** | **Definition** |
| --- | --- |
| High | We are very confident that the true effect lies close to that of the estimate of the effect. |
| Moderate | We are moderately confident in the effect estimate: The true effect is likely to be close to the estimate of the effect, but there is a possibility that it is substantially different |
| Low | Our confidence in the effect estimate is limited: The true effect may be substantially different from the estimate of the effect. |
| Very low | We have very little confidence in the effect estimate: The true effect is likely to be substantially different from the estimate of effect. |

**Supplementary Table 4. Risk of bias assessment of cross-sectional studies: Newcastle–Ottawa Scale (adapted version)**

| **Source** | **Selection** | | | | **Comparability based on design and analysis** | **Outcome** | | **Total** | **Assessment** |
| --- | --- | --- | --- | --- | --- | --- | --- | --- | --- |
|  | **Representativeness of the sample** | **Sample size** | **Non-respondents** | **Ascertainment of the exposure** |  | **Assessment of outcome** | **Statistical test** |  |  |
| Sonoda C.,2018 | 1 | 1 | 0 | 2 | 1 | 2 | 1 | 8 | Good |
| Pham T.A.V., 2018 | 1 | 1 | 0 | 2 | 2 | 2 | 1 | 9 | Very Good |
| Martinez-Herrera M., 2017 | 1 | 1 | 0 | 2 | 1 | 2 | 1 | 8 | Good |
| Kim Y.-S., 2017 | 1 | 1 | 0 | 2 | 2 | 2 | 1 | 9 | Very Good |
| Goulart A.C., 2017 | 0 | 1 | 0 | 2 | 2 | 2 | 1 | 8 | Good |
| Kitagawa M., 2017 | 1 | 1 | 0 | 2 | 2 | 2 | 1 | 9 | Very Good |
| Habashneh R.A., 2016 | 1 | 1 | 1 | 2 | 1 | 2 | 1 | 9 | Very Good |
| Benguigui C., 2012 | 1 | 1 | 0 | 2 | 2 | 2 | 1 | 9 | Very Good |
| Pataro A.L., 2012 | 1 | 1 | 1 | 2 | 2 | 2 | 1 | 10 | Very Good |
| Katagiri S., 2010 | 1 | 1 | 1 | 2 | 1 | 2 | 1 | 9 | Very Good |
| Shimazaki Y., 2010 | 1 | 1 | 0 | 2 | 2 | 2 | 1 | 9 | Very Good |
| Han D.-H., 2010 | 1 | 1 | 1 | 2 | 2 | 2 | 1 | 10 | Very Good |
| Ikbariyeh B. 2021 | 1 | 1 | 0 | 2 | 2 | 2 | 1 | 9 | Very Good |
| Al-Zahrani et al., 2003 | 1 | 1 | 0 | 2 | 2 | 2 | 1 | 9 | Very Good |
| Borges-Yáñez et al., 2006 | 1 | 1 | 1 | 2 | 2 | 2 | 1 | 10 | Very Good |
| D'Aiuto et al., 2008 | 1 | 1 | 0 | 2 | 2 | 2 | 1 | 9 | Very Good |
| Dalla Vecchia et al., 2005 | 1 | 1 | 0 | 2 | 2 | 2 | 1 | 9 | Very Good |
| Ekuni et al., 2008 | 1 | 1 | 0 | 2 | 1 | 2 | 1 | 8 | Good |
| Furuta et al., 2010 | 1 | 1 | 0 | 2 | 1 | 2 | 1 | 8 | Good |
| Haffajee; Socransky., 2009 | 1 | 1 | 0 | 2 | 2 | 2 | 1 | 9 | Very Good |
| Khader et al., 2009 | 1 | 1 | 0 | 2 | 2 | 2 | 1 | 9 | Very Good |
| Han et al., 2009 | 1 | 1 | 1 | 2 | 2 | 2 | 1 | 10 | Very Good |
| Wang et al., 2009 | 1 | 1 | 0 | 2 | 1 | 2 | 1 | 8 | Good |
| Torrungruang et al., 2005 | 1 | 1 | 0 | 2 | 1 | 2 | 1 | 9 | Very Good |
| Kongstad et al., 2009 | 1 | 1 | 1 | 2 | 2 | 2 | 1 | 10 | Very Good |
| Kumar et al., 2009 | 1 | 1 | 1 | 2 | 0 | 2 | 1 | 8 | Good |
| Kushiyama et al.,2009 | 1 | 1 | 1 | 2 | 2 | 2 | 1 | 10 | Very Good |
| Morita et al., 2009 | 1 | 1 | 1 | 2 | 2 | 2 | 1 | 10 | Very Good |
| Saito et al., 2001 | 1 | 1 | 1 | 2 | 0 | 1 | 1 | 7 | Good |

** For cross-sectional studies, Very good quality: 9-10 points, Good quality: 7-8 points, Satisfactory quality: 5-6 points, Unstatisfactory quality: 0-4 points

**Supplementary Table 5. Assessment of the risk of bias of the cohort studies: Newcastle–Ottawa Scale**

| **Study** | **Selection** | | | | **Comparability based on design and analysis** | **Outcome** | | | **Total** | **Assessment** |
| --- | --- | --- | --- | --- | --- | --- | --- | --- | --- | --- |
|  | **Representativeness of sample** | **Selection of non-intervention cohort** | **Ascertainment of exposure** | **Proof that outcome of interest was not evident at pointt of study** |  | **Assessment of outcome** | **Follow-up long enough for outcomes to arise** | **Adequacy of follow-up of cohorts** |  |  |
| Lee J.-H., 2017 | 0 | 1 | 1 | 1 | 2 | 1 | 1 | 0 | 7 | Good |
| Chen T.-P. et al 2021 | 1 | 1 | 1 | 1 | 2 | 1 | 1 | 1 | 9 | Good |
| Morita T. 2019 | 1 | 1 | 1 | 1 | 2 | 1 | 1 | 0 | 8 | Good |
| Machado et al., 2005 | 0 | 1 | 1 | 1 |  | 1 | 1 | 0 | 5 | Fair |

* For cohort studies, Good quality: 3 or 4 points in selection domain AND 1 or 2 points in compatibility domain AND 2 or 3 points in outcome/exposure domain, Fair quality: 2 points in selection domain AND 1 or 2 points in comparability domain AND 2 or 3 points in outcome/exposure domain, Poor quality: 0 or 1 point in selection domain OR 0 points in comparability domain OR 0 or 1 point in outcome/exposure domain

**Supplementary Table 6. Assessment of risk of bias of the case-control studies: Newcastle–Ottawa Scale**

| **Study** | **Selection** | | | | **Comparability based on design and analysis** | **Outcome** | | | **Total** | **Assessment** |
| --- | --- | --- | --- | --- | --- | --- | --- | --- | --- | --- |
|  | **Is the case definition adequate?** | **Patients are representative** | **Selection of Controls** | **Definition**  **of Controls** |  | **Assessment of exposure** | **Same method of ascertainment for patients and controls** | **Non-response rate** |  |  |
| Marro F. 2021 | 1 | 1 | 1 | 1 | 2 | 1 | 1 | 0 | 8 | Good |
| Dumitrescu; Kawamura., 2010 | 1 | 1 | 1 | 1 | 1 | 1 | 1 | 0 | 7 | Good |
| Saxlin et al. 2010 | 1 | 1 | 1 | 1 | 2 | 1 | 1 | 0 | 8 | Good |
| Saito, 2008 | 0 | 0 | 1 | 1 | 2 | 1 | 1 | 0 | 6 | fair |

* For case-control studies, Good quality: 3 or 4 points in selection domain AND 1 or 2 points in compatibility domain AND 2 or 3 points in outcome/exposure domain, Fair quality: 2 points in selection domain AND 1 or 2 points in comparability domain AND 2 or 3 points in outcome/exposure domain, Poor quality: 0 or 1 point in selection domain OR 0 point in comparability domain OR 0 or 1 point in outcome/exposure domain
